# Anti-Allergic Effect of Low Molecular Weight Digest from Abalone Viscera on Atopic Dermatitis-Induced NC/Nga

**DOI:** 10.3390/md19110634

**Published:** 2021-11-12

**Authors:** Tae-Hee Kim, Seong-Yeong Heo, Gun-Woo Oh, Won Sun Park, Il-Whan Choi, Zhong-Ji Qian, Won-Kyo Jung

**Affiliations:** 1 Department of Biomedical Engineering and New-Senior Healthcare Innovation Center (BK21 Plus), Pukyong National University, Busan 48513, Korea; taehee94@pukyong.ac.kr; 2 Marine Integrated Biomedical Technology Center, The National Key Research Institutes in Universities, Pukyong National University, Busan 48513, Korea; hsyadsl@naver.com (S.-Y.H.); ogwchobo@naver.com (G.-W.O.); 3 Research Center for Marine Integrated Bionics Technology, Pukyong National University, Busan 48513, Korea; 4 Jeju Marine Research Center, Korea Institute of Ocean Science & Technology (KIOST), Jeju 63349, Korea; 5 Department of Physiology, Kangwon National University School of Medicine, Chuncheon 24341, Korea; parkws@kangwon.ac.kr; 6 Department of Microbiology, Inje University College of Medicine, Busan 48516, Korea; cihima@inje.ac.kr; 7 School of Chemistry and Environmental Science, College of Food Science and Technology, Guangdong Ocean University, Zhanjiang 524088, China; zjqian@gdou.edu.cn; 8 Southern Marine Science and Engineering Guangdong Laboratory, Zhanjiang 524088, China

**Keywords:** atopic dermatitis, abalone viscera, *Haliotis discus hannai*, gastrointestinal digest, *Dermatophagoides farina*

## Abstract

Abalone viscera (AV) is one of the byproducts of the seafood processing industry. The low molecular weight (<5 kDa) peptides (LMW-AV) obtained from gastrointestinal digestion of AV could suppress allergenic responses on activated HMC-1 human mast cells in our previous study. Regarding the allergenic response of LMW-AV, in the present study, we further investigated the potential of oral administration of LMW-AV against atopic dermatitis (AD) in a dermatitis-induced model stimulated with *Dermatophagoides farinae*. The results demonstrated that the LMW-AV reduced a number of clinical symptoms, such as the severity of the dermatitis and serum immunoglobulin E levels. Moreover, LMW-AV could inhibit the expression of chemokines and cytokines. The histological analysis indicated that the LMW-AV has suppressed the eosinophil count and the mast cell infiltration into the upper dermis. The results suggest that LMW-AV can be considered as a promising candidate for AD treatment.

## 1. Introduction

Pacific abalone, *Haliotis discus hannai*, is an edible shellfish and a worldwide popular seafood due to its high nutritional value with respect to high protein, essential amino acids and fatty acids content against its wet mass [1]. Abalone consists of numerous bioactive substances to exert anti-inflammatory, antioxidant, and anti-tumor effects [2,3,4]. Due to its pharmacological values, the consumption of abalone has increased and its production has increased simultaneously by 60-fold over the past 10 years, especially in Wando Island, southwestern coast of South Korea, which is a major mariculture area for this species [4].

Abalone viscera (AV) weighs 15–25% of total body weight and is generally considered as one of the main byproducts of the mass scale abalone processing industry [5]. To increase utilization of AV, it is supplied as porridge, source, and simple cooked food in the market, yet not as functional foods, because their pharmaceutical activities and underlying mechanisms of AV had rarely been reported. Therefore, many researchers have recently investigated their biological activities and responsible cellular signaling mechanisms [3,6,7]. However, the anti-allergic activity of AV has not been reported sufficiently to use it as a potential agent against allergic reactions.

Naturally derived bioactive peptides have been reported with numerous biological activities including osteoblast differentiation, antioxidant, and anti-allergic activities [5]. In vitro gastrointestinal (GI) digestion is an effective way to obtain protein hydrolysate containing bioactive peptides [8]. Among various natural resources, marine organisms have gained increasing attention to obtain hydrolysates since they have been reported with various biological activates [6,7,9].

Atopic dermatitis (AD) is a chronic inflammatory skin disorder characterized by skin lesions and hypersensitivity that is mainly caused by environmental, psychological, immunological, and genetic factors [10,11]. It generally presents with mild to severe erosion, itching, skin rash, edema, an unpleasant sensation associated with the desire to scratch, and pustules from secondary bacterial infection and is frequently accompanied by a variety of inflammatory skin conditions and systemic diseases [12,13]. Topical glucocorticoids have been used extensively for AD treatment, but they have been reported to have several side effects, such as skin atrophy, increased bruising, telangiectasia, tachyphylaxis, and cutaneous irritation [14]. Therefore, recent research has identified candidates for AD treatment from marine organisms and investigated their preventive effect on AD as alternative therapeutic agents.

Furthermore, our previous study revealed that fractionated AV hydrolysates have an inhibitory effect on allergic reaction in activated HMC-1 human mast cells and low molecular weight digest from abalone viscera (LMW-AV) has the highest anti-allergic activity among fractionated AV hydrolysates [6]. After purification and identification, a nonameric peptide (AIGDP, PFNQGTFAS, MW: 1,175 Da) was isolated from LMW-AV, and then it was revealed that AIGDP attenuates allergic reaction in activated HMC-1 human mast cells. Thus, we hypothesized that LMW-AV as therapeutic agent would exhibit an anti-allergic effect on an AD mice model stimulated with *Dermatophagoides farinae* (DfE).

## 2. Results

### 2.1. Preparation and Composition of LMW-AV

LMW-AV were prepared using gastrointestinal enzymes in a sequential order; pepsin (Phase I) and trypsin and α-chymotrypsin (Phase II). The degree of hydrolysis (DH) was measured as 74% after the Phase I digestion and DH was finally measured as 92% after the Phase II enzymatic digestion (Table 1). After fractionation using an ultrafiltration (UF) membrane bioreactor system, the yield of LMW-AV was measured as 26.4%. The proximate analysis of live AV showed 70.5 ± 0.7% (moisture), 23.1 ± 1.3% (crude protein), 1.3 ± 0.2% (crude lipid), 3.2 ± 0.4% (carbohydrate), and 3.1 ± 0.4% (ash) on a live-weight basis (Table 2). Due to the presence of a high amount of protein, 23.1 ± 1.3% in AV, there could be a greater potential to have therapeutically active peptides. The amino acid composition of AV and LMW-AV is summarized in Table 3. AV was rich in acidic acids (glutamic acid and glutamine, 4.62% of AV powder and aspartic acid and asparagine, 1.16% of AV powder), which together constituted 5.78% of AV. LMW-AV also was rich in acidic acids (glutamic acid, 16.17% and aspartic acid, 5.96%), which together constituted 22.13% of the total amino acid residues. Moreover, LMW-AV overall increased the percentage of amino acid residues due to in vitro GI digestion and has significantly increased the composition of proline and alanine higher than these of the AV.

### 2.2. Inhibitory Effects of LMW-AV on the Development of Dermatitis in NC/Nga Mice

**Figure 1:**
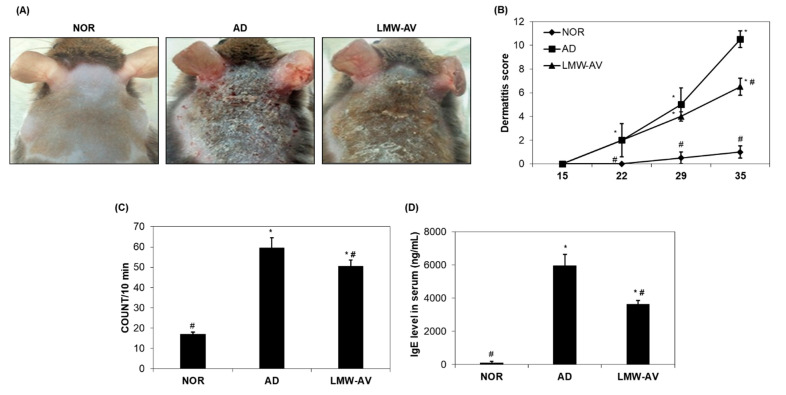
Effects of LMW-AV on development of dermatitis. (**A**) Pictures of the clinical observations on the back of mice. (**B**) Evaluation of clinical dermatitis induced by Biostir-AD ointment. (**C**) Scratching behavior was assessed on the last day of the study (day 35). (**D**) Effects of LMW-AV on serum IgE level. Each column shows the mean ± SD of 5 mice. * *p* < 0.05 indicates significant differences compared with the NOR group and ^#^ *p* < 0.05 indicates significant differences compared with the AD group.

We investigated the anti-atopic activity of LMW-AV in AD-induced NC/Nga mice using a modified SCORAD index once a week after the first application of Biostir-AD ointment for a total of three weeks. There was no effect on body weight or other obvious adverse effects noticed by the oral administration of LMW-AV throughout the experimental period (Supplementary Materials, Figure S1). Superficial dermatitis was not observed in the untreated (NOR) group throughout the experimental period. In contrast, the AD group exhibited progressive symptoms, including edema, erythema, scaling, and excoriation (Figure 1A). Administration of LMW-AV significantly suppressed the AD-like skin lesions on the dorsal surface. All mice in the AD group exhibited increasing AD-like skin lesions and dermatitis scores depending on the number of times that the Biostir-AD ointment was applied (Figure 1B). However, the administration of LMW-AV decreased the dermatitis scores; particularly significant reduction was observed after 35 days.

We also monitored scratching behavior for 10 min after 34 days. Figure 1C shows that scratching behavior was higher in AD and LMW-AV groups than in the NOR group, but the spontaneous scratching in the LMW-AV group was lower than that in the AD group. These consistent results indicated that the administration of LMW-AV attenuates the clinical AD symptoms induced by the application of Biostir-AD ointment.

### 2.3. Measurement of Serum Immunoglobulin E (IgE)

We investigated whether LMW-AV also regulates the levels of serum IgE in AD mice. Application of the Biostir-AD ointment significantly increased serum IgE levels, but the LMW-AV group significantly decreased the levels of AD-induced serum IgE compared to the AD group (Figure 1D). This result indicates that the administration of LMW-AV suppresses serum IgE levels, thus contributing to the inhibition of the development of AD-like skin.

### 2.4. Inhibitory Effects of LMW-AV on mRNA Expression of Chemokines and Cytokines Induced by AD-like Lesions

**Figure 2:**
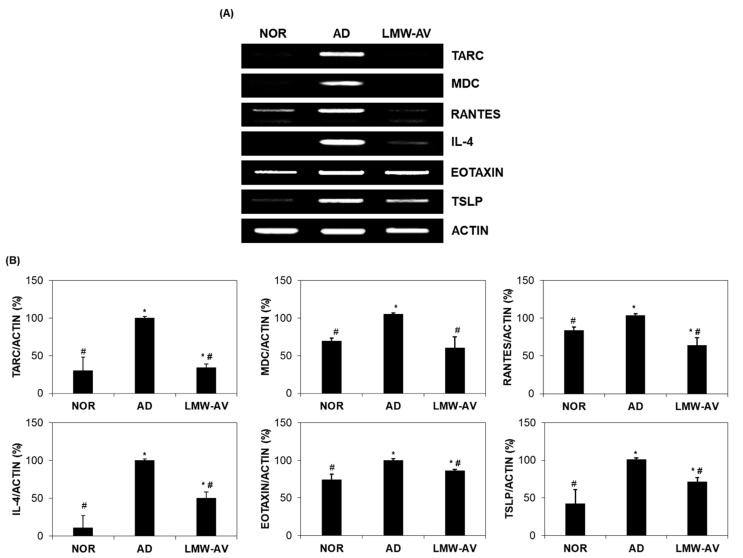
Effect of the LMW-AV on mRNA expression of cytokine and several chemokines on AD-like skin lesions stimulated with Biostir-AD ointment. (**A**) Gel images and (**B**) graphs of relative mRNA expression level of cytokines and chemokines were obtained by RT-PCR analysis. Actin mRNA expression levels were used to confirm the equal amounts of RNA used for cDNA synthesis. Each column shows the mean ± SD of five mice. * *p* < 0.05 indicates significant differences compared with the NOR group and ^#^ *p* < 0.05 indicates significant differences compared with the AD group.

We examined the effect of LMW-AV on the mRNA expression of chemokines and cytokines associated with the pathogenesis of AD, including eotaxin, interleukin (IL)-4, macrophage-derived chemokine (MDC), regulated upon activation, normal T cell expressed and presumably secreted (RANTES), thymic stromal lymphopoietin (TSLP), and thymus and activation regulated chemokine (TARC), in dorsal skin lesions of AD-induced mice. As shown in Figure 2, mRNA expression of type-2 chemokines and cytokines was significantly higher in the AD group while significantly lower in the LMW-AV administrated group. These results indicate that LMW-AV alleviates AD symptoms by inhibiting the mRNA expression of Th2-related chemokines and cytokines in AD-like skin lesions

### 2.5. Histological Observation of the Dorsal Skin Tissue

**Figure 3:**
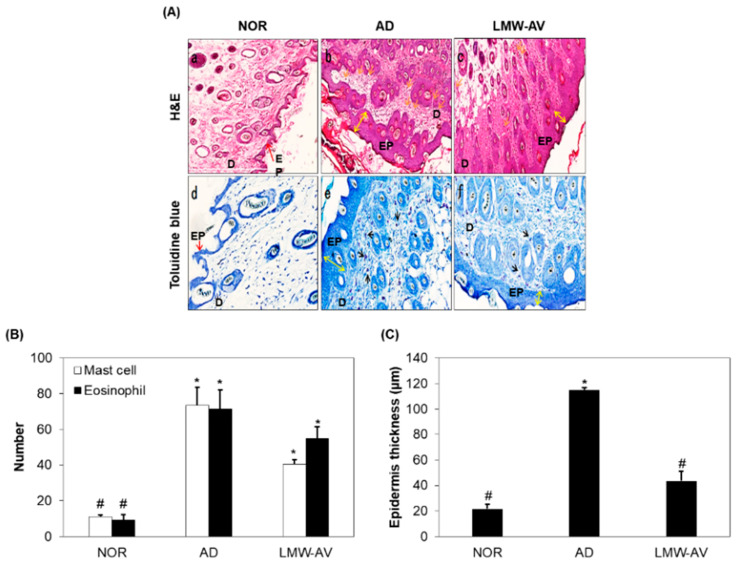
Effect of the LMW-AV on AD-induced skin. (**A**) Histopathological analysis of the dorsal area of the skin. Histopathological analysis of dorsal tissue. (**a**–**c**) Slide sections of dorsal tissue were evaluated by staining with H&E staining and observed at 100× magnification. (**d**–**f**) Slide sections of dorsal tissue were evaluated by staining with 0.25% toluidine blue and observed at 200× magnification. The yellow arrows indicate skin thickness of epidermis, the yellow arrow indicates epidermis, the black arrows indicate mast cells in epidermis and dermis, and the orange arrows indicate eosinophil in dermis. (**B**) The number of infiltrated mast cells per specific area was measured using toluidine blue-stained section and eosinophils per specific area was measured using H&E staining image. (**C**) Epidermis thickness per specific area was measured. Data are presented as mean ± SD (n = 5). * *p* < 0.05 indicates significant differences compared with the NOR group and ^#^ *p* < 0.05 indicates significant differences compared with the AD group.

To investigate the anti-allergic effect of LMW-AV, histological analysis was conducted using hematoxylin-eosin (H&E) and toluidine blue staining (Figure 3A). H&E staining revealed that the thickness of the epidermis and the dermis in AD group was greater than that in the NOR group due to edema and the accumulation of inflammatory cells, indicating a severe condition (Figure 3C). However, oral administration of LMW-AV reduced the thickness of the skin and edema (Figure 3(Aa–Ac)). Toluidine blue staining revealed the excessive infiltration of mast and eosinophil into the upper dermis within the AD group. However, LMW-AV suppressed the infiltration of mast cells into the epidermis compared to the AD group (Figure 3(Ad–Af)). Moreover, the number of mast cells and eosinophils in the dorsal skin was also counted and the results are presented in Figure 3B. The application of Biostir-AD ointment led to a significant increase in the number of mast cells (73.5 ± 10.0) and eosinophils (114.91 ± 1.71) in the upper dermis; in contrast, the administration of LMW-AV decreased the number of mast cells by 38% (43.5 ± 2.5) and eosinophils by 50% (55.0 ± 6.45) compared to the AD group. Overall, the histological analysis found that the oral administration of LMW-AV suppresses AD-like histological changes by reducing edema, and decreasing the number of eosinophils, the infiltration of mast cells, and increasing epidermal thickness.

## 3. Discussion

AD is a T cell-mediated hypersensitivity and inflammatory disease characterized by Th1/Th2 imbalance, IgE hypersensitivity, and clinical symptoms such as chronic pruritus, red to brownish-gray itchy patches, and eczematous skin lesions [15,16]. T cells play a crucial role in immune responses where the number of CD4^+^ T cells increases in the AD-like skin lesions [16,17]. The activated CD4^+^ T cells can be divided into Th1 and Th2 cells depending on the secreted cytokines [18]. Th2 cytokines, such as IL-4, IL-5, IL-13, are dominant over Th1 cytokines, such as tumor necrosis factor-α (TNF-α) and IL-1β, during the acute phase of AD [14,19]. Th2 cytokines are predominantly produced by CD4^+^ T cells and mast cells and are also thought to be central to the pathogenesis of AD due to their ability to regulate IgE synthesis [20,21]. Th2 chemokines are thought to repeatedly induce percutaneous sensitization by upregulating allergen entry through the impaired skin barrier [22]. In particular, eotaxin is involved in the pathogenesis of the acute AD phase via allergen sensitization and is involved in the activation of eosinophils [23,24]. TSLP is an IL-7-like cytokine that is found at significantly higher levels in both acute and chronic skin lesions of AD [25,26]. TARC and MDC are cytokines produced by several cells, such as keratinocytes, dendritic cells, and platelets, and have been associated with the severity of AD [27,28]. In particular, TARC is produced by dermal dendritic cells after stimulation by TSLP and controls the migration of Th2 cells into the skin [27]. RANTES is a chemokine produced by keratinocytes that is important in terms of the cutaneous inflammation of AD and is involved in the activation and migration of eosinophils and T cells [29,30]. For these reasons, inhibiting the expression of these chemokines and cytokines represents a promising approach to the prevention of skin diseases including AD.

In vitro GI digestion using digestive enzymes such as pepsin, trypsin, and α-chymotrypsin can be used in the production of absorbable bioactive peptides that show resistance to physiological digestion after oral administration [2,8,31]. Furthermore, the peptides produced by GI digestion have increased stability and bioaccessibility compared to non-GI digestion which can be more easily hydrolyzed by peptidases of the microbial flora in the intestine [32]. Hence, in the present study, we prepared in vitro GI digestion of *H. discus hannai* viscera, which is discarded as a byproduct from the food processing industry and investigated the potentials in anti-allergic activity. In particular, low molecular weight peptides derived from marine organism show higher pharmacological activity than high molecular weight peptides [33,34]. Therefore, these facts supported that LMW-AV has effective anti-allergic activity compared to other molecular weight fractions.

AIGIDs and/or isolated peptides from AIGIDs have reported a diverse range of biological activities, including anti-allergic, anti-inflammatory, anti-matrix metalloproteinase, and osteogenic activities [2,3,7,35]. Despite their biological activities, to the best of our knowledge, no previous study has reported on their anti-allergic activity and the mechanism of action based on in vivo experiments. However, we decided to carry out in vivo studies using LMW-AV, due to the low yield in the purification of AIGDP (<1%) and the high amount required for oral administration to animals.

First, we analyzed amino acids composition of AV and LMW-AV. After in vitro GI digestion, the ratios of two amino acids (Ala and Pro) relatively increased compared to those of AV. Additionally, Glu maintained the highest ratio in LMW-AV, as well as AV. According to previous studies, three major amino acids (Ala, Glu, and Pro) in LMW-AV are known as major amino acids modulating immune function by regulating immune cell functions [36,37]. Furthermore, our previous study separated three fractions using fast protein liquid chromatography and purified the nonameric peptide (AIGDP) from the biggest fraction for the highest yield [6]. Based on these results, we hypothesized that the high ratios of three amino acids, as well as AIGDP, can mediate the relief of AD.

House dust mites, including DfE, are the most common environmental factor associated with AD [38]. DfE increases serum IgE levels and the development of AD skin lesions, including edema, hemorrhage, erosion, and dryness. NC/Nga mice, which are usually used as the animal model for human clinical AD cases, spontaneously develop AD-like skin lesions with an elevation of serum IgE levels [39,40,41]. For these reasons, in our present study, we selected DfE-induced NC/Nga mice to investigate the anti-allergic effect of LMW-AV.

We investigated the anti-allergic activity of LMW-AV for AD-induced NC/Nga mice through in vivo experiments. The oral administration of LMW-AV suppressed AD-like clinical symptoms and decreased scratching frequency and dermatitis scores compared with the AD group. Serum IgE levels were observed to increase after the application of Biostir-AD ointment, but these levels were much lower in the LMW-AV group. To examine the mRNA expression of Th2-related chemokines and cytokines, we conducted reverse transcriptase polymerase chain reaction (RT-PCR) analysis. The AD group exhibited a significant increase in the mRNA expression of Th2-related chemokines and cytokines, while the oral administration of LMW-AV significantly decreased their levels. Although the inhibitory effect of LMW-AV was not statistically significant in histological analysis, it also showed that the administration of LMW-AV reduced the number of eosinophil and mast cells and their infiltration into the upper dermis. However, histological analysis for only mast cells and eosinophils in AD-induced NC/Ngc model biopsy is the major limitation of this study. Therefore, further studies need to be conducted using different animal models and histological analyses on basophils and neutrophils in addition to mast cells and eosinophils

## 4. Materials and Methods

### 4.1. Materials

Live adult abalones (*H. discus hannai*) were collected from Wando Island, Wando-gun, Korea. After washing abalones with tap water, AV was separated and lyophilized until used. Mouse IgE enzyme-linked immunosorbent assay (ELISA) kit was purchased from BD Biosciences (San Jose, CA, USA). Biostir-AD ointment including mite allergens derived from DfE was purchased from Biostir (Hiroshima, Japan). Chloroform, eosin, hematoxylin, isopropyl alcohol, and toluidine blue were purchased from Sigma-Aldrich (St. Louis, MO, USA). TRIzol reagent was purchased from Invitrogen (Carlsbad, CA, USA). Ketamine was purchased from Yuhan (Seoul, Korea) and rompun was purchased from Bayer Healthcare (Kyunggi-do, Korea). Other common analytical-grade chemicals and reagents used in this study were commercially available.

### 4.2. Preparation of In Vitro GI Digestion and Fractionation on a UF Membrane Bioreactor System

In vitro GI digestion was performed according to the previously described method [9]. One hundred milliliters of 4% (*w/v*) AV solution was adjusted to pH 2.2 in gastric digestion (phase I) using 1 M HCl and 1 M NaOH while being vigorously mixed. Pepsin was added at an E/S ratio of 1/250 (*w/w*) and then incubated at 37 °C in a shaker. After 2 h, the pH was set to 6.5 to mimic the conditions of intestinal digestion (phase II). Similarly, trypsin and α-chymotrypsin were both supplemented at an E/S ratio of 1/250 (*w/w*). The solution was further incubated at 37 °C for 2.5 h. When the samples were taken at the beginning and end of digestion, the pH was adjusted to 8.0. The samples were centrifuged at 10,000× *g* for 15 min at 4 °C, and the supernatant was lyophilized to obtain a dry powder of LMW-AV. DH of LMW-AV following GI digestion steps was measured using DNS assay [42]. The LMW-AV was fractionated using an UF membrane bioreactor system with MW cut-offs (MWCOs) of 5 kDa and those recovered from the fractionation were lyophilized.

### 4.3. Analysis of Proximate Composition

The moisture, ash, protein, and lipid contents of AV were determined using the AOAC method with some modifications [33], and the carbohydrate content was determined by the phenol-sulfuric acid method [43].

### 4.4. Amino Acid Composition

For the determination of amino acid compositions, the lyophilized AV and LMW-AV (20 mg of powder) were hydrolyzed in 6 M HCl containing 0.1% thioglycolic acid at 110 °C for 24 h under vacuum. Amino acid compositions were identified and quantified using an automatic amino acid analyzer (Biochrom 20; Pharmacia Biotech, Uppsala, Sweden).

### 4.5. Animal

Male NC/Nga mice (specific pathogen-free) aged 7 weeks were purchased from Orient Bio Inc. (Seoul, Korea) and housed in a specific pathogen-free animal facility. All experimental procedures were approved by the institutional animal care and utilization committee of Pukyong National University (Number PKNUIACUC-2018-07). The mice were maintained under specific pathogen-free conditions at 24 ± 2 °C, 55 ± 10% relative humidity, and 12 h/12 h light/dark cycle. Mice were provided with a commercial pelleted feed (5L79, Orient Bio, Seongnam, Korea) and autoclaved water ad libitum during the experiments.

### 4.6. Animal Experimental Design

After 1 week of acclimation, mice were randomly divided into the following three groups (n = 5 per group): untreated (NOR group), only application of 100 mg Biostir-AD ointment (AD group), application of 100 mg Biostir-AD ointment, and oral administration of LMW-AV at a dose of 50 mg/kg/day (LMW-AV group).

**Figure 4:**
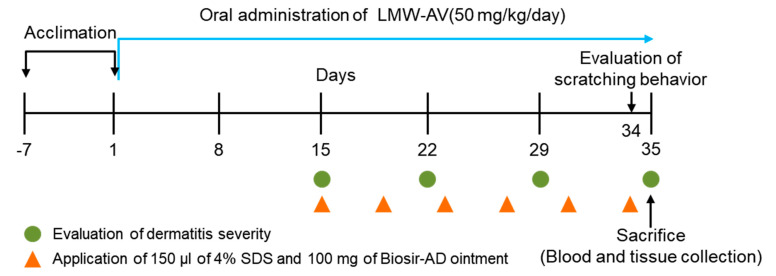
Schematic diagram of the study protocol. The experimental scheme for induction of AD-like skin lesions in NC/Nga mice by application of Biostir-AD ointment and oral administration of LMW-AV.

All surgical procedures were performed under aseptic conditions. All mice were anesthetized during the operation using intraperitoneal injections of combination solution of ketamine, rompun, and phosphate-buffered saline (a ratio of 2:2:7, respectively) and the dorsal skin surface was shaved. To disrupt the skin barrier, 150 μL of 4% (*w/v*) sodium dodecyl sulfate was topically applied to the shaved dorsal skin surface 3 h before Biostir-AD ointment application. Biostir-AD ointment was applied twice/week for 3 weeks to induce AD-like skin lesions. To investigate the preventive effect of LMW-AV on AD-like skin lesions in NC/Nga mice stimulated with Biostir-AD ointment, LMW-AV was orally administrated to mice using an oral-zoned needle connected to a 1 mL syringe at a dose of 50 mg/kg/day for 5 weeks (from 2 weeks before the first application of Biostir-AD ointment). A flow chart of the animal experiment is shown in Figure 4.

### 4.7. Evaluation of Dermatitis Severity and Body Weight

The body weight of mice was measured once a week. The severity of AD-like dorsal skin lesions was assessed by a modified SCORing AD method once/week for 3 weeks after the first application of Biostir-AD ointment. The degree of each symptom, such as erythema/hemorrhage, scarring/dryness, edema, excoriations/erosion, was scored as 0 (absence), 1 (mild), 2 (moderate), and 3 (severe). Clinical skin score, defined as the sum of the individual scores, ranged from 0 to 12. The dorsal skin of each mouse was photographed before sacrifice. Dermatitis severity was evaluated by 2 independent observers.

### 4.8. Evaluation of Scratching Behavior

Scratching behavior was measured before 1 day from sacrifice. After an acclimation period of at least 1 h, scratching behavior was measured for 10 min. Scratching of the AD-like lesions of the dorsal region by the hind paws was counted and that of other regions was disregarded. Scratching behavior was evaluated by 2 independent observers.

### 4.9. Measurement of Serum IgE Level

Blood samples were collected after mice were sacrificed from all groups. After the blood had clotted at room temperature, serum samples were obtained by centrifugation at 7000× *g* for 10 min at 4 °C and were stored at −70 °C until use. The serum IgE level was measured using a mouse IgE ELISA kit according to the manufacturer’s instructions. Then, the absorbance was measured at 450 nm using an EL800^®^ microplate reader (BioTek Instruments, Inc., Winooski, VT, USA).

### 4.10. RT-PCR

The mRNA expression level of chemokines and cytokines in the AD-like lesion was determined by RT-PCR analysis. After sacrifice, the dorsal skins were homogenized and were treated with TRIzol reagent. The suspension was mixed with chloroform and centrifuged at 13,000× *g* for 10 min at 4 °C. The aqueous phase was precipitated by mixing with isopropyl alcohol and centrifuged at 13,000× *g* for 10 min at 4 °C. The precipitate was washed with 75% ethanol, dried, and then dissolved in diethyl pyrocarbonate (DEPC) containing water. The total RNA content was calculated based on the absorbance at 260 nm and the quality was determined at absorbance ratio of 260/280 nm. Equal amounts of RNA (1 μg) were used for each cDNA synthesis reaction using the Maxime RT-Premix kit according to the manufacturer’s instruction (iNtRON Biotechnology, Seoul, Korea). Then, cDNA was amplified using Accupower PCR Premix (Bioneer, Daejeon, Korea). PCR was performed using primers for eotaxin, IL-4, MDC, RANTES, TARC, TSLP, and actin. The sequences of primers are described in Table 4. The following PCR conditions were applied for all amplifications: 30 cycles of 95 °C for 30 s (denaturing), 55 °C for 45 s (annealing), and 72 °C for 45 s (primer extension). Following amplification, portions of the PCR reactions were electrophoresed on a 3% agarose gel for 20 min as 100 V. The gels were visualized after staining with ethidium bromide (EtBr) using UNOK-8000 Gel Manager System (Biotechnology, Seoul, Korea) and mRNA expression was quantified by Image J software (National Institutes of Health, Bethesda, MD, USA).

### 4.11. Histological Analysis

Standard histological paraformaldehyde fixation, paraffin embedding, and histological staining were performed. Briefly, the skin biopsy samples were fixed in 4% paraformaldehyde for 24 h and then embedded in paraffin. Sections of the skin (5 μm) were stained with H&E or toluidine blue to monitor the histological changes in the skin and recruitment of mast cells and eosinophils, respectively. All stained slides were scanned using a Hamamatsu digital slide scanner (Hamamatsu, Japan) and images were obtained using the Hamamatsu NonoZoomer Digital Pathology solution. The number of infiltrated mast cells and eosinophils in dermis, and epidermis thickness of randomly selected areas of each animal were measured by the same histologists, who were blinded to the groups. Measurement of infiltrated mast cell number was conducted using the toluidine blue-stained section and measurement of eosinophil number was conducted using the H&E-stained section.

### 4.12. Statistical Analysis

All data are presented as the mean ± standard deviation (SD) of at least three independent experiments. The statistical significance of the differences observed between groups was assessed by a non-parametric Kruskal–Wallis test. All statistical analyses were performed using the SPSS Statistics 27.0 software (SPSS, Inc., Chicago, IL, USA). * *p* < 0.05 was considered statistically significant compared with only the NOR group. ^#^ *p* < 0.05 were considered statistically significant compared with only the AD group.

## 5. Conclusions

In the present study, we demonstrated that LMW-AV extract from *H. discus hannai* significantly ameliorates clinical AD symptoms. These effects were determined to be mediated via the inhibition of mRNA expression of Th2-related chemokines and cytokines in AD-like skin lesions. These consistent results suggest that LMW-AV should be considered a promising candidate for AD treatment. These consistent results suggest that LMW-AV has great potential as a promising agent for the treatment of AD with minimal side effects.

## Figures and Tables

**Table 1:** Digestion condition and degree of hydrolysis.

GI Digestion Steps	Enzyme/Substrate(E/S) Ratio (Enzyme)	pH	Temp. (°C)	Incubation Time (h)	DH (%)
Gastric digestion (Phase I)	1:250 (Pepsin)	2.2	37	12	74
Intestinal digestion (Phase II)	1:250 (Tyrpsin, α-chymotrypsin)	6.5	37	8	92

**Table 2:** Proximate composition in AV (%).

Proximate Composition	Moisture	Protein	Lipid	Carbohydrate	Ash
Abalone viscera	70.5 ± 0.7	23.1 ± 1.3	1.3 ± 0.2	3.2 ± 0.4	3.1 ± 0.4

**Table 3:** Contents of compositional amino acids in AV and LMW-AV.

Amino Acid	AV (%)	LMW-AV (%)
Asp/n ^1^	1.16	5.96
Thr	0.56	4.65
Ser	0.58	2.38
Glu/n ^2^	4.62	16.17
Pro	0.41	14.04
Gly	0.86	5.68
Ala	0.53	10.55
Cys	0.19	4.40
Val	0.51	4.58
Met	0.22	0.50
Ile	0.34	3.54
Leu	0.61	6.99
Tyr	0.4	2.66
Phe	0.41	3.79
Trp	0.02	1.76
His	0.31	4.97
Lys	0.54	4.90
Arg	0.41	2.50
Total	12.68	100

^1^ Asp/n: aspartic acid + asparagine, ^2^ Glu/n: glutamic acid + glutamine.

**Table 4:** Primer sequences for RT-PCR.

Genes	Forward	Reverse
Eotaxin	5′ CCA AGG ACT TGG CTT CAT GTA G 3′	5′ ATT CTG GCT TGG CAT GGT AGC 3′
IL-4	5′ TCA TCG GCA TTT TGA ACG AGG T 3′	5′ GCA TCG AAA AGC CCG AAA GAG 3′
MDC	5′ TCT GAT GCA GGT CCC TAT GGT 3′	5′ TTA TGG AGT AGC TTC TTC AC 3′
RANTES	5′ ATC ATC CTC ACT GCA GCC GC 3′	5′ CAC ACT TGG GGG TTC CTT CG 3′
TARC	5′ CAG GAA GTT GGT GAG CTG GTA TA 3′	5′ TTG TGT TCG CCT GTA GTG CAT A 3′
TSLP	5′ CTG TAC TGT TAA TGA CCA GC 3′	TCG TAG ATG AAG GCT CT 3′
Actin	5′ TTG GCA ATG AGC GGT TCC 3′	5′ AGC ACT GTG TTG GCG TAC 3′

## Data Availability

Not applicable.

## Supplementary Materials

The following are available online at https://www.mdpi.com/article/10.3390/md19110634/s1. Figures S1: Body weights in each group of NC/Nga mice.
