# Supplementary material for: Anti-Allergic Effect of Low Molecular Weight Digest from Abalone Viscera on Atopic Dermatitis-Induced NC/Nga
Source: Mar Drugs. 2021 Nov 12;19(11):634. doi: 10.3390/md19110634 (PMC8618959; doi:10.3390/md19110634)
Supplement: Supplementary file 1 [file marinedrugs-19-00634-s001.zip › marinedrugs-1421368-supplementary.pdf]

Electronic Supplementary Information

Anti-allergic Effect of Low Molecular Weight Digest from Abalone Viscera on Atopic Dermatitis-Induced NC/Nga

Tae-Hee Kim [1,3,#], Seong-Yeong Heo [2,3,#], Gun-Woo Oh [2,3], Won Sun Park [4], Il-Whan Choi [5], Zhong-Ji Qian [6,7] and Won-Kyo Jung [1,2,3,*]

[1] Department of Biomedical Engineering and New-senior Healthcare Innovation Center (BK21 Plus), Pukyong National University, Busan 48513, Republic of Korea

[2] Research Center for Marine Integrated Bionics Technology, Pukyong National University, Busan 48513, Republic of Korea

[3] Marine integrated Biomedical Technology center, The National Key Research Institutes in Universities, Pukyong National University, Busan 48513, Republic of Korea

[4] Department of Physiology, Kangwon National University School of Medicine, Chuncheon, 24341, Republic of Korea

[5] Department of Microbiology, Inje University College of Medicine, Busan, 48516, Republic of Korea

[7] Southern Marine Science and Engineering Guangdong Laboratory, Zhanjiang 524-088, China

[6] School of Chemistry and Environmental Science, Guangdong Ocean University, College of Food Science and Technology, Zhanjiang 524-088

* Correspondence: wkjung@pknu.ac.kr (W.-K.J.); Tel.: Tel.: +82-51-629-5775

# These authors contributed equally to this study

List of Figures

Figure S1. Body weights in each group of NC/Nga mice were monitored once/week for 3 weeks after the first application of Biostir-AD ointment. Values shows the mean ± SD of 5 mice.

Measure of body weights

Body weights of the NC/Nga mice were monitored once a week during the experimental period.

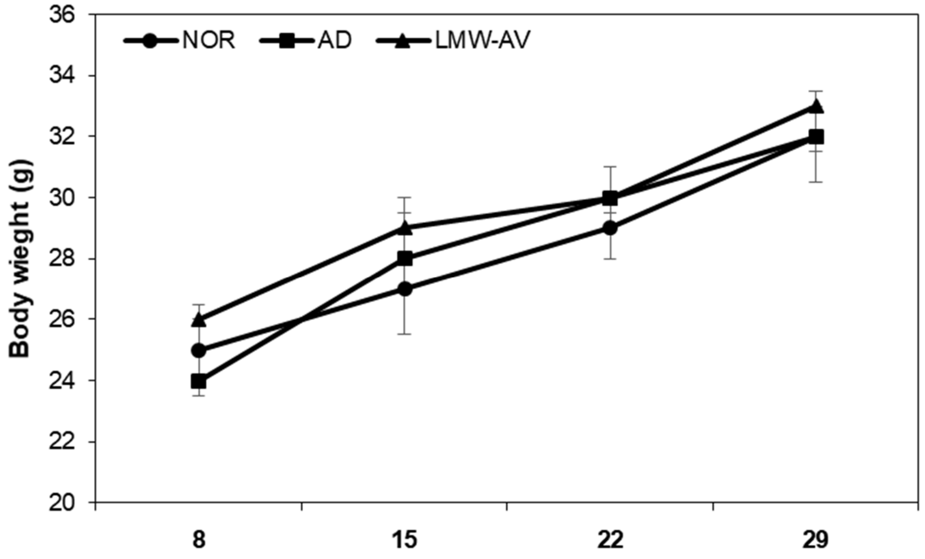

Figure S1. Body weights in each group of NC/Nga mice were monitored once/week for 3 weeks after the first application of Biostir-AD ointment. Values shows the mean ± SD of 5 mice.
